# Fast Open-Source Toolkit for Water T2 Mapping in the Presence of Fat From Multi-Echo Spin-Echo Acquisitions for Muscle MRI

**DOI:** 10.3389/fneur.2021.630387

**Published:** 2021-02-26

**Authors:** Francesco Santini, Xeni Deligianni, Matteo Paoletti, Francesca Solazzo, Matthias Weigel, Paulo Loureiro de Sousa, Oliver Bieri, Mauro Monforte, Enzo Ricci, Giorgio Tasca, Anna Pichiecchio, Niels Bergsland

**Affiliations:** ^1^Division of Radiological Physics, Department of Radiology, University Hospital of Basel, Basel, Switzerland; ^2^Department of Biomedical Engineering, University of Basel, Allschwil, Switzerland; ^3^Advanced Imaging and Radiomics Center, Neuroradiology Department, IRCCS Mondino Foundation, Pavia, Italy; ^4^Translational Imaging in Neurology (ThINk) Basel, Department of Biomedical Engineering, University Hospital Basel and University of Basel, Allschwil, Switzerland; ^5^Neurologic Clinic and Policlinic, Departments of Medicine, Clinical Research and Biomedical Engineering, University Hospital Basel and University of Basel, Basel, Switzerland; ^6^ICube, Université de Strasbourg, Centre National de la Recherche Scientifique (CNRS), Strasbourg, France; ^7^Unità Operativa Complessa di Neurologia, Fondazione Policlinico Universitario A. Gemelli IRCCS, Rome, Italy; ^8^Dipartimento di Neuroscienze, Istituto di Neurologia, Università Cattolica del Sacro Cuore, Rome, Italy; ^9^Department of Brain and Behavioral Sciences, University of Pavia, Pavia, Italy; ^10^Department of Neurology, Buffalo Neuroimaging Analysis Center, Jacobs School of Medicine and Biomedical Sciences, University at Buffalo, The State University of New York, Buffalo, NY, United States; ^11^Fondazione Don Carlo Gnocchi Onlus (IRCCS), Milan, Italy

**Keywords:** MRI, neuromuscular diseases, relaxometry, free open source software, water T2 relaxation time, fat water imaging

## Abstract

Imaging has become a valuable tool in the assessment of neuromuscular diseases, and, specifically, quantitative MR imaging provides robust biomarkers for the monitoring of disease progression. Quantitative evaluation of fat infiltration and quantification of the T2 values of the muscular tissue's water component (wT2) are two of the most essential indicators currently used. As each voxel of the image can contain both water and fat, a two-component model for the estimation of wT2 must be used. In this work, we present a fast method for reconstructing wT2 maps obtained from conventional multi-echo spin-echo (MESE) acquisitions and released as Free Open Source Software. The proposed software is capable of fast reconstruction thanks to extended phase graphs (EPG) simulations and dictionary matching implemented on a general-purpose graphic processing unit. The program can also perform more conventional biexponential least-squares fitting of the data and incorporate information from an external water-fat acquisition to increase the accuracy of the results. The method was applied to the scans of four healthy volunteers and five subjects suffering from facioscapulohumeral muscular dystrophy (FSHD). Conventional multi-slice MESE acquisitions were performed with 17 echoes, and additionally, a 6-echo multi-echo gradient-echo (MEGE) sequence was used for an independent fat fraction calculation. The proposed reconstruction software was applied on the full datasets, and additionally to reduced number of echoes, respectively, to 8, 5, and 3, using EPG and biexponential least-squares fitting, with and without incorporating information from the MEGE acquisition. The incorporation of external fat fraction maps increased the robustness of the fitting with a reduced number of echoes per datasets, whereas with unconstrained fitting, the total of 17 echoes was necessary to retain an independence of wT2 from the level of fat infiltration. In conclusion, the proposed software can successfully be used to calculate wT2 maps from conventional MESE acquisition, allowing the usage of an optimized protocol with similar precision and accuracy as a 17-echo acquisition. As it is freely released to the community, it can be used as a reference for more extensive cohort studies.

## Introduction

Neuromuscular disorders encompass genetic and acquired diseases of lower motor neurons, peripheral nerves, neuromuscular junction, or skeletal muscle, generally causing different degrees of motor impairment in the affected patients. In particular, muscular dystrophies are hereditary degenerative disorders of skeletal muscles, causing progressive replacement of muscle tissue by fat. This happens through disparate pathological processes and molecular mechanisms that are, at least to some extent, disease-specific and related to the peculiar genetic defect that characterizes each of them (1).

However, some of these broad pathological processes are shared by most muscular dystrophies. They can be followed on muscle imaging as an early phase of muscle damage and intramuscular edema, often corresponding to inflammatory/necrotic changes (2, 3), and subsequent stages characterized by progressive deposition of fat and connective tissue (4–6). Different pathological processes are generally present simultaneously in the same patient or even in the same muscle group.

The good soft tissue characterization capabilities of MRI can be exploited to quantify the status of these ongoing processes in the muscle; for example, global T2 contrast can be used to highlight edema, and fat/water separation methods can show fat infiltration.

When moving toward quantitative imaging, global T2, extracted by a monoexponential fitting is a sensitive disease indicator when fat infiltration is not present. However, in neuromuscular diseases where adipocytes significantly replaced muscular tissue, it highly correlates with the fat content of the muscle. In this case, it is therefore rather an indicator of long-term changes in the musculature, albeit an indirect one with respect to fat fraction quantification; conversely, the T2 relaxation of the water component of the tissue (wT2) well correlates with acute “disease activity” (7).

Various acquisition methods have been proposed to quantify wT2 independently of fat infiltration (8–15). In current clinical practice, multi-echo spin-echo (MESE) sequences are typically used, and various types of exponential fitting (biexponential, triexponential) are used for T2 calculation (16, 17). However, these methods are sensitive to multiple confounding factors, such as B1 inhomogeneities.

Marty et al. (14) presented a method based on an extended phase graph (EPG) fitting that addresses many of these issues. Besides, in contrast to other methods such as the ones proposed by Klupp et al. (12), Sousa et al. (11), or Koolstra et al. (13), the EPG-based approach has the advantage of using a conventional spin-echo sequence for the quantification, which is broadly available and therefore does not require any particular sequence modification or hardware. In recent years, this method has been extensively used for wT2 quantification in several studies (18–22). Although there are small differences in the exact protocol used depending on the MR scanner vendor, the differences in the implementation of the fitting process can be more substantial.

As a result of the EPG fitting, a relatively precise estimation of the fat fraction is also obtained. The resulting fat fractions approximate the results of a three-point Dixon acquisition, which has been proven to differentiate well between patients of various neuromuscular diseases and healthy subjects (16, 23). However, fat fraction estimation is not the primary quantitative target of this method, and it can be an issue when accurate fat fraction maps are needed, as weighting factors [such as magnetization transfer effects in multi-slice MESE acquisitions (24–26)] can bias the estimation. An alternative approach to obtain this information is to use a dedicated sequence (usually based on small flip-angle multi-echo gradient-echo imaging) for a purely proton-density-weighted fat fraction estimation. This approach also allows using more accurate fat models that incorporate the complex chemical composition of fat (27). In practice, a typical muscle MRI examination usually comprises a dedicated volume acquisition for single muscle identification (segmentation) and for accurate fat fraction acquisition.

In this work, we build upon this concept to present a fast and open software for wT2 fitting, which can be used as a reference implementation for reproducibility studies. The postprocessing performance is optimized by the usage of GPU processing and the creation of a dictionary (of adjustable size) of simulated signals incorporating slice profile information (14, 28). Additionally, this work further extends the initial concept as it implements multiple fitting methods for the wT2 estimation, and it can incorporate the information coming from a separate fat/water acquisition (of arbitrary resolution and field of view) to constrain the fitting. With this constraint, the possibility of reducing the number of acquired echoes for the fitting (thus reducing the scan time) is also analyzed.

## Materials and Methods

### Software Implementation

A stand-alone, command-line application was developed in Python, using standard mathematical extension libraries (NumPy, SciPy) and, for hardware acceleration, the pycuda extension for interfacing with the general-purpose graphical processing unit.

The core of the application consists of a fast, GPU-accelerated implementation of signal simulation based on the extended phase graph concept (29, 30). As the method is intended for multi-echo spin-echo acquisitions, a Carr-Purcell-Meiboom-Gill (CPMG) (31, 32) simulation is performed at every run of the program, adapting the timing and the number of echoes to the actual sequence parameters. The slice profile of the radiofrequency pulses is taken into account in the simulation, assuming hanning-windowed sinc pulses. The slice profile is calculated through the application of a Shinnar-LeRoux transform (33) and the slice width of the refocusing pulse is assumed to be a factor of 1.2 larger than the excitation pulse, as per characteristics of the pulse sequence of the used MR scanner. For different vendors and acquisition protocols, the user can provide custom slice profiles and refocusing width factors as parameters to the program. The simulation logic is directly written in GPU-specific C++ language for maximum efficiency. Thanks to the high parallelization of the GPU tasks, signals corresponding to multiple combinations of wT2, fat fraction, and B1 inhomogeneity factors can be simulated concurrently and placed in a dictionary, whose size can be chosen by the user. The fat model is assumed to have a single spectral peak with a fixed T2 value, which can either be given *a priori* or estimated from the input data. T1 values for both water and fat are assumed to be constant (1,400 and 365 ms, respectively), as in (14).

Subsequently, the time course of each voxel in the input series is compared to each entry in the dictionary by using the correlation metric. The parallelization of the GPU is again leveraged in this step, as the correlation operation is equivalent to the following matrix multiplication:

$$\begin{array}{l} \text{C}=\text{S}\times \text{D} \end{array}$$

where **S** is the signal matrix, having a separate voxel on each row and time in the column direction; **D** is the dictionary matrix, having time in the row direction and dictionary entries in the row directions; **C** is the result matrix holding the signal correlations with the dictionary. After multiplication, the index of the maximum value of each column identifies the parameter combination that best fits the measured signal.

The above procedure describes the unconstrained fitting. When an externally derived fat fraction map is also provided, it is aligned (based on the slice orientation and position) with each slice of the original spin-echo data. The information is then taken into account by restricting the maximum search to the given fat fraction for each voxel.

In addition to EPG fitting, the software can also perform double-exponential fitting. This fitting can neither correct for B1 inhomogeneity nor slice profile and is meant to be used when the EPG fitting is unstable or the true slice profile is not known.

The code is released under a free software license (GNU General Public License v3) at the website: https://www.github.com/fsantini/MyoQMRI.

### Acquisition Protocol

A conventional 2D multi-slice multi-echo spin-echo (MESE) acquisition protocol was prepared for a commercial whole-body 3T MR scanner (MAGNETOM Skyra, Siemens Healthcare, Erlangen, Germany) equipped with an 18-channel body array coil and integrated spine coil with the following acquisition parameters: number of echoes 17, number of slices 7, TR 4,100 ms, first TE and echo spacing 10.9 ms, bandwidth 250 Hz/px, matrix size 192 × 384, resolution 1.2 × 1.2 mm^2^, slice thickness 10 mm, gap between slices 30 mm.

For fat fraction quantification, a 3D multi-echo gradient-echo (MEGE) acquisition using a custom sequence was prepared with the following parameters: number of echoes 6, TR 35 ms, first TE/echo spacing 1.7/1.5 ms, flip angle 7°, bandwidth 1,050 Hz/px, matrix size 396 × 432 × 52, resolution 1.0 × 1.0 × 5.0 mm3. The sequence had a monopolar readout with interleaved echo spacing (even and odd echoes acquired in subsequent repetitions). The acquired images were postprocessed with the publicly-available algorithm FattyRiot (27) to obtain the fat fraction maps.

### Human Experiments

The acquisitions described above were performed on the thighs of 5 (three male, median age 46 y, range 29–61 y) subjects with diagnosed facioscapulohumeral muscular dystrophy (FSHD), which is a particular form of muscular dystrophy characterized by progressive muscle wasting and fatty replacement often preceded by muscle edema on MRI (34), and of four subjects without a history of neuromuscular diseases (two male, median age 54.5 y, range 26–72 y). The acquisitions were performed according to the local ethics regulations and informed consent was obtained from the participants.

### Data Analysis

The computer used for the postprocessing is a current mid-range personal computer (Ryzen 2600, Advanced Micro Devices, Santa Clara, CA, equipped with 32GB of RAM and a GeForce 1060 GPU, NVIDIA corporation, Santa Clara, CA).

The proposed algorithm was applied to the MESE acquisition after generation of a dictionary containing 60 linearly spaced values for wT2 (range 20–80 ms), 20 values for B1 factor (range 40–140%), and 101 values for the fat fraction (range 0–100%), for a total of 121,200 parameter combinations. The fat T2 was estimated from a subsample of the subjects (through a single-component EPG fitting in regions of subcutaneous fat) and subsequently assumed constant at 151 ms. Maps derived from EPG and double exponential fitting, with and without the constraint of the external proton-density-weighted fat fraction as calculated from the MEGE acquisition, were produced (an overview of the assumed and fitted parameters for each method is given in Table 1). The fitting was repeated by discarding later spin echoes (and only retaining 8, 5, or 3) to evaluate the robustness of the algorithm with respect to the number of echoes.

**Table 1:** Overview of the presented fitting methods.

**Method**	**Constrained variables**	**Fitted parameters**
EPG unconstrained	Water T1, Fat T1, Fat T2, Slice profile	wT2, Fat Fraction, B1
EPG constrained	Water T1, Fat T1, Fat T2, Slice profile, Fat Fraction (voxelwise from MEGE)	wT2, B1
Double exponential unconstrained	Fat T2	wT2, Fat Fraction
Double exponential constrained	Fat T2, Fat Fraction (voxelwise from MEGE)	wT2

Regions of interest (ROIs) were manually drawn by one reader bilaterally on every MESE slice over the cross section of the following muscles: Vastus Lateralis (VM), Vastus Medialis (VM), Rectus Femoris (RF).

The average and standard deviation for wT2 values and fat fraction were extracted, and the following indicators were calculated:

- Average error between fat fraction calculated from MESE and the one deriving from MEGE (considered the “gold standard”) - only for nonconstrained reconstructions, calculated as:
  $$\begin{array}{l} ERR_{ff}=\underset{pop,subj,roi}{\text{mean}}\left(FF_{MESE}\right)-\underset{pop,subj,roi}{\text{mean}}\left(FF_{MEGE}\;\right), \end{array}$$
- where $\underset{pop,subj,roi}{\text{mean}}$ represents the averaging operation over the whole population, over all ROIs of each subject, and over the voxels of each ROI.
- Pooled standard deviation across the ROIs—this indicator is similar to an “average of the standard deviations,” and it indicates the variability of the fitted wT2 values over a small spatial region; it is related to noise or tissue inhomogeneities due to disease activity and is calculated as follows:
  $$\begin{array}{l} SD_{pooled}=\sqrt{\underset{pop,subj}{\text{mean}}\left(\underset{roi}{{\text{sd}}^{\text{2}}}\left(T_{2}\right)\;\right)}, \end{array}$$
- where $\underset{roi}{{\text{sd}}^{\text{2}}}$ represents the squared standard deviation over each ROI.
- Standard deviation of the averages over the ROIs (hereby termed “intrasubject standard deviation”) - this indicator relates to the variability over larger areas, for example arising from field inhomogeneity, and is calculated as follows:
  $$\begin{array}{l} SD_{intrasubject}=\sqrt{\underset{pop}{\text{mean}}\left(\underset{subj}{{\text{sd}}^{\text{2}}}\left(\underset{roi}{\text{mean}}\left(T_{2}\right)\right)\right)}\; \end{array}$$
- This indicator was only calculated in the volunteer cohort, because patients might have variability in the wT2 values due to their pathology.
- Global average wT2 for patients and control groups, calculated as follows:
  $$\begin{array}{l} {\text{mean}}_{T2,(patients|controls)}=\underset{patients|controls}{\text{mean}}\left(\underset{subj,roi}{\text{mean}}\left(T_{2}\right)\right)\\ \;{\text{sd}}_{T2,(patients|controls)}=\underset{patients|controls}{\text{sd}}\left(\underset{subj,roi}{\text{mean}}\left(T_{2}\right)\right) \end{array}$$

Statistical analysis was performed with R (35). To quantify the influence of the fat fraction on the fitting of wT2, the correlation between the average fat fraction and the average wT2 for each ROI was calculated. For the calculation of the correlation coefficients the log-transformed fat fractions were used to compensate for the skewness of the fat fraction distribution. However, the visualization was done on the original fat fraction axis (0–100%) for ease of interpretation.

In addition, correlation of repeated measurements (rmcorr function) instead of the standard Pearson coefficient was used to compensate for dependence of measurements of the same subject. In this case the subject is introduced as a variable and both wT2 and fat fraction are considered as measures.

### Validation

In order to evaluate the absolute accuracy of the fitting method, the same data was analyzed using the EPG wT2 fitting procedure of the QMRTools software package (36). The slice profile was adapted to match the one used in the current acquisitions and the fitting was applied to the full 17-echo dataset.

The same ROIs as in the previous analysis were considered, and the average wT2 in each ROI was calculated and compared to the corresponding ROI of our method. Average and standard deviation of the errors were calculated, and the agreement was visualized in a Bland-Altman plot.

## Results

The software fitted the MESE datasets with EPG dictionary matching in an average time of 2 s/slice, plus 12 s per dataset for the generation of the dictionary (operation performed at every run of the program, which was faster than caching the results to disk). The double exponential fitting was not GPU-optimized and took 390 s (6 m 30 s) per slice.

**Figure 1:**
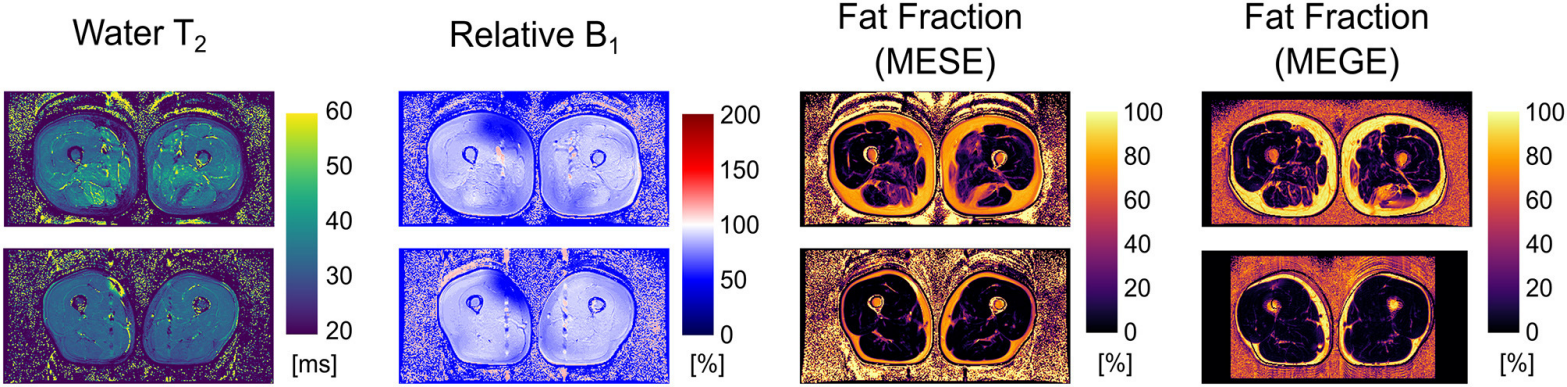
Parameter maps derived from an unconstrained 17-echo EPG dictionary matching for an FSHD patient (**top row**) and a healthy volunteer (**bottom row**). The rightmost panel is the registered fat fraction map deriving from a multi echo gradient echo acquisition (MEGE) for reference.

Exemplary outputs from an unconstrained 17-echo reconstruction for a patient and a volunteer are shown in Figure 1. No noticeable artifacts are visible in the wT2 and fat fraction maps, whereas the B1 maps show some inconsistencies, mostly located in the fascia, thus remaining mostly masked in the other maps.

**Figure 2:**
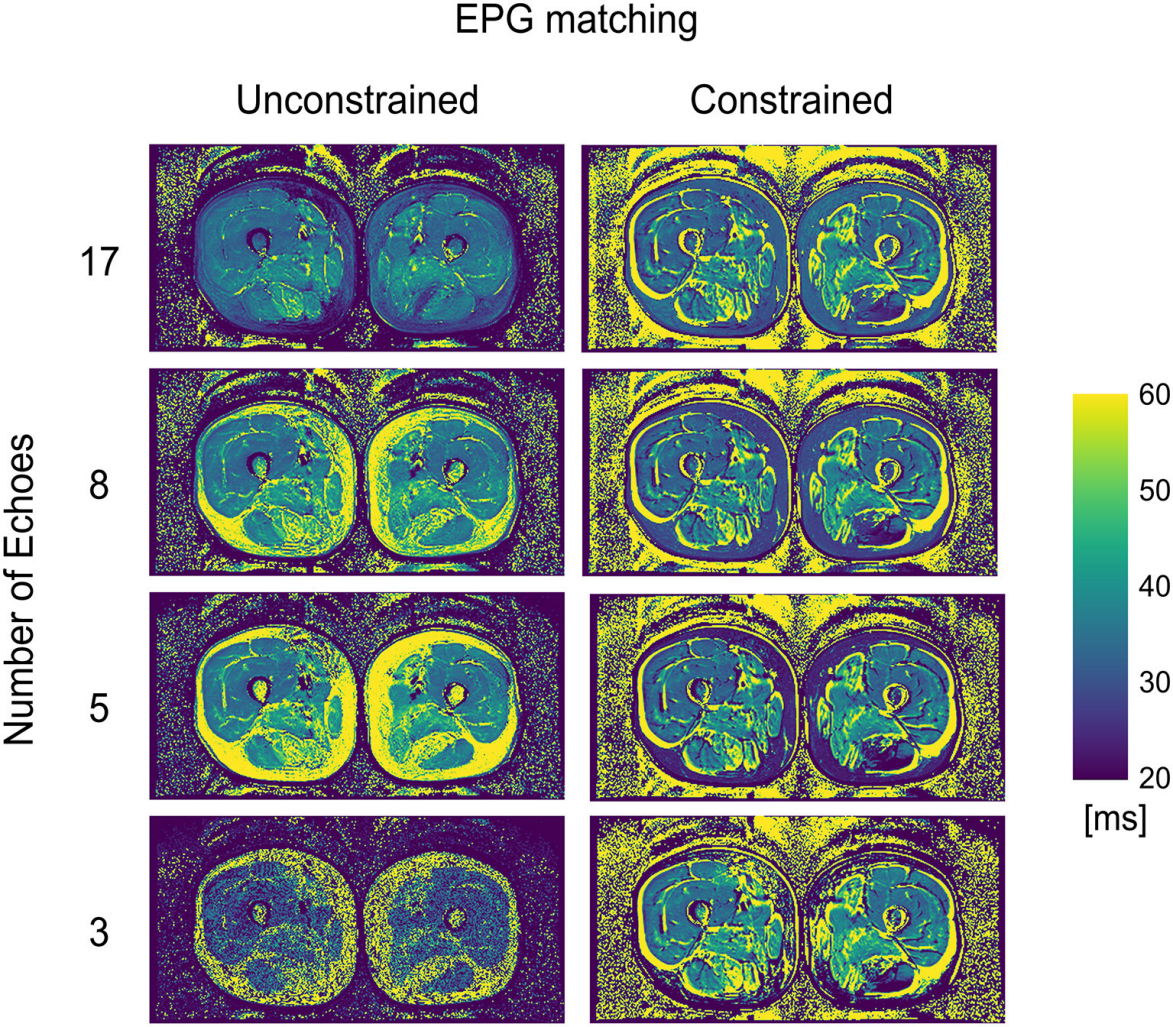
wT2 maps for an FSHD patient using a varying number of acquired echoes by EPG simulation with and without an external fat fraction map as a reconstruction constraint.

**Figure 3:**
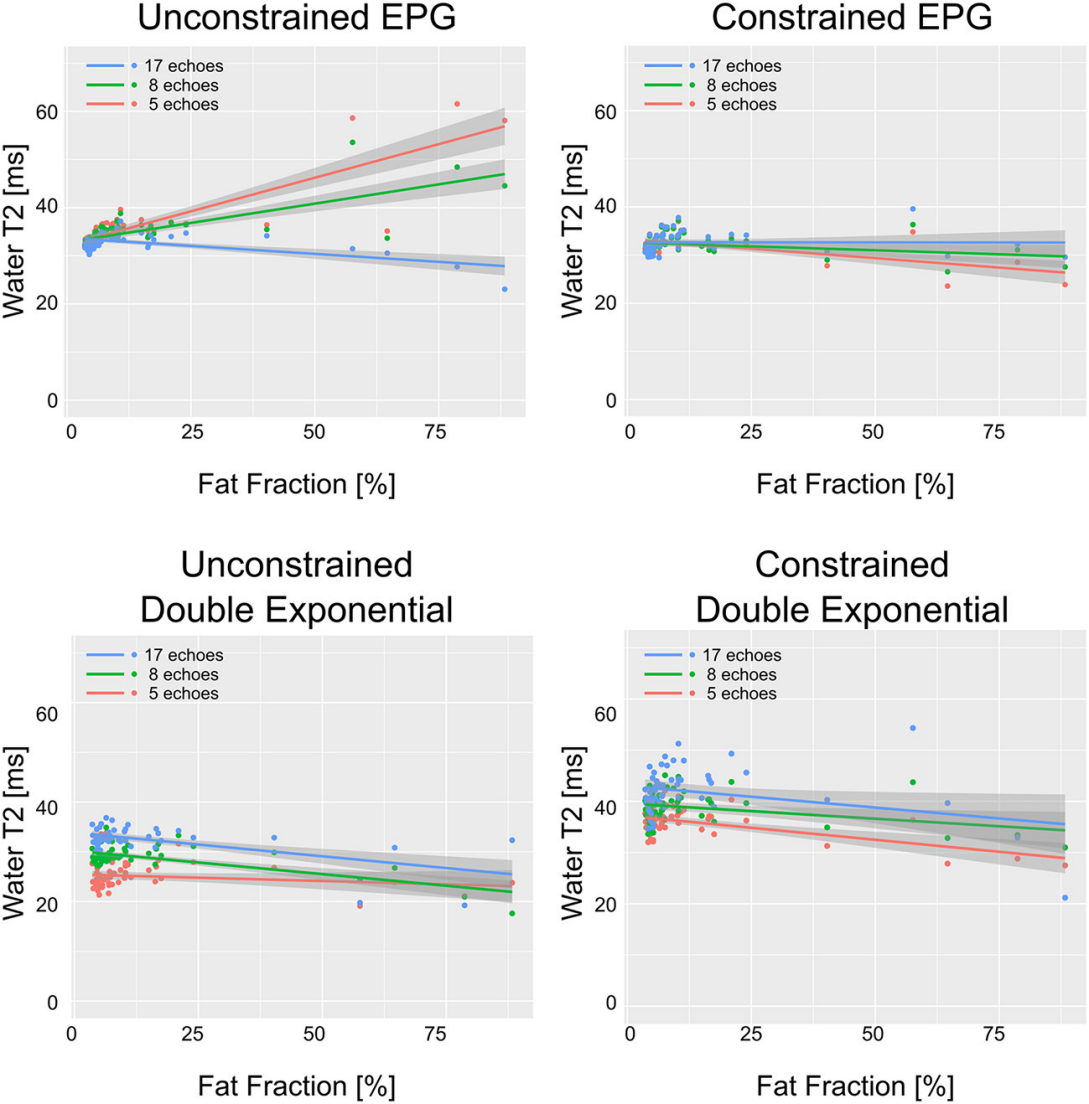
wT2 values over each ROI vs. fat fraction in the same ROI, for all ROIs drawn in patients and healthy volunteers. The solid lines represent the linear regression with shaded gray areas indicating the 95% confidence intervals.

WT2 maps had a homogeneous appearance with EPG matching both for 17 and 8 echoes (Figure 2). However, the unconstrained reconstruction showed a noticeable difference in quantitative values in areas with heavy fat infiltration, whereas the fat-fraction-constrained reconstructions appeared similar between 17, 8, and 5 considered echoes. The visual quality of the map was insufficient in any combination when only three echoes were used for the reconstruction (Figure 2). Quantitatively, it can be observed that a reduced number of echoes in the unconstrained fitting has an effect on wT2 fitting when fat infiltration is present, resulting in a highly significant positive correlation (*p* < 0.001) for EPG fitting with 5 and 8 echoes (correlation coefficient *r* = 0.73 and 0.61, respectively, Figure 3). Conversely, the correlation was significantly negative (*r* = −0.71, *p* < 0.001) for the unconstrained fitting with 17 echoes, and for the constrained fitting of 5 and 8, but not 17 echoes (*r* = −0.60, −0.41, −0.19; *p* < 0.001, *p* = 0.005, 0.2, respectively).

**Figure 4:**
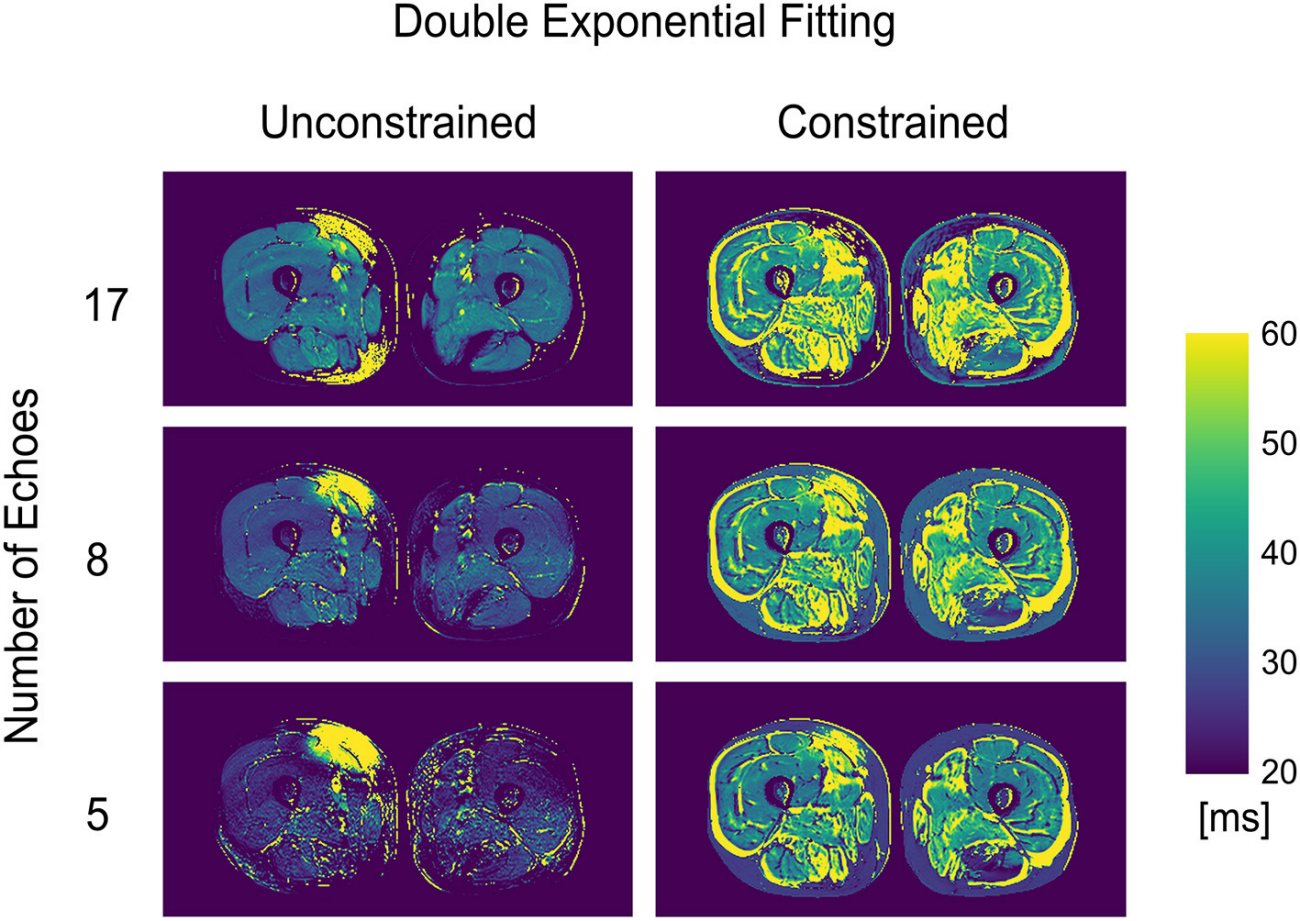
wT2 maps for an FSHD patient using a varying number of acquired echoes by double exponential fitting with and without an external fat fraction map as a reconstruction constraint.

The double exponential fitting showed decreased wT2 with any number of echoes when unconstrained fitting was used, in addition to artifacts arising from B1 inhomogeneities (see Figure 4 and Table 2). Correlation between wT2 and the fat fraction was always negative and significant (*p* = 0.02 for the constrained 8 and 17 echoes, *p* < 0.001 otherwise) in all cases except, notably, the unconstrained fitting of five echoes (*p* = 0.29), with negative correlation coefficients ranging from *r* = −0.34 for the constrained 8-echo reconstruction to *r* = −0.62 for the unconstrained 8-echo reconstruction (Figure 3).

**Table 2:** Summary of results for the different fitting methods, with and without an external proton-density-weighted fat fraction (FF) constraint.

**Method**	**FF Constraint**	**# echoes**	**wT2 (ms)**							**FF (%)**		**Global FF** **error (p.p.)**
			**Volunteers**				**Patients**			**Vol**.	**Pat**.	
			**Average**	**Global SD**	**Pooled SD**	**Intrasubject SD**	**Average**	**Global SD**	**Pooled SD**			
EPG	No	3	28.6	1.3	8.1	1.0	31.4	6.2	12	17.3	22.5	6.4
		5	34.2	1.8	5.2	1.1	37.7	7.7	8.8	2.9	8.1	−8.0
		8	33.8	1.8	4.4	1.0	36.1	4.9	7.6	3.2	9.8	−7.0
		17	33.0	1.3	3.3	0.9	32.7	2.5	4.1	4.6	12.8	−4.7
	Yes	3	34.5	1.5	5.9	1.5	34.5	3.6	8.9	6.4^*^	17.8^*^	
		5	32.3	1.3	4.9	1.1	32.2	3.2	6.4			
		8	31.9	1.4	4.9	1.3	32.4	2.6	5.9			
		17	32.1	1.6	5.1	1.4	33.1	2.5	6.2			
Double exponential	No	5	25.3	2.9	8.3	3.1	24.7	2.5	10.1	17.9	23.9	8.6
		8	29.4	1.7	5.8	1.8	28.4	3.1	6.7	15.1	21.9	6.2
		17	33.2	1.6	4.6	1.6	31.7	3.7	9.5	11.9	19.0	3.2
	Yes	5	36.1	2.1	6.9	2.3	35.4	3.6	8.3	6.4^*^	17.8^*^	
		8	38.6	2.5	7.7	2.7	38.4	3.7	9.3			
		17	41.3	3.5	10.4	3.5	41.2	6.2	14			

The pooled standard deviation (SD) is associated with image noise, whereas the intrasubject SD is associated with homogeneity in different anatomical regions. The FF error with respect to the multi-echo gradient-echo acquisition (MEGE) is given in percentage points (p.p.). Values marked with an asterisk (*) are not fitted by the algorithm but are derived from the MEGE acquisition.

According to the quantitative quality metrics (Table 2), considering a higher number of echoes improved both the noise (pooled standard deviation) and the homogeneity across the ROIs for each subject (intrasubject variability). For a reduced number of echoes, the constrained reconstruction retained good homogeneity down to five considered echoes for the EPG reconstruction (3.1 ms for the constrained case vs. 6.7 for the unconstrained). The double exponential fitting showed good homogeneity (intrasubject standard deviation ranging from 2.7 to 4.6 milliseconds), but more noise (pooled standard deviation ranging from 5.9 to 10.5 ms).

The constrained reconstruction resulted in a generally higher pooled standard deviation due to the artifacts introduced by the misregistration of the images.

**Figure 5:**
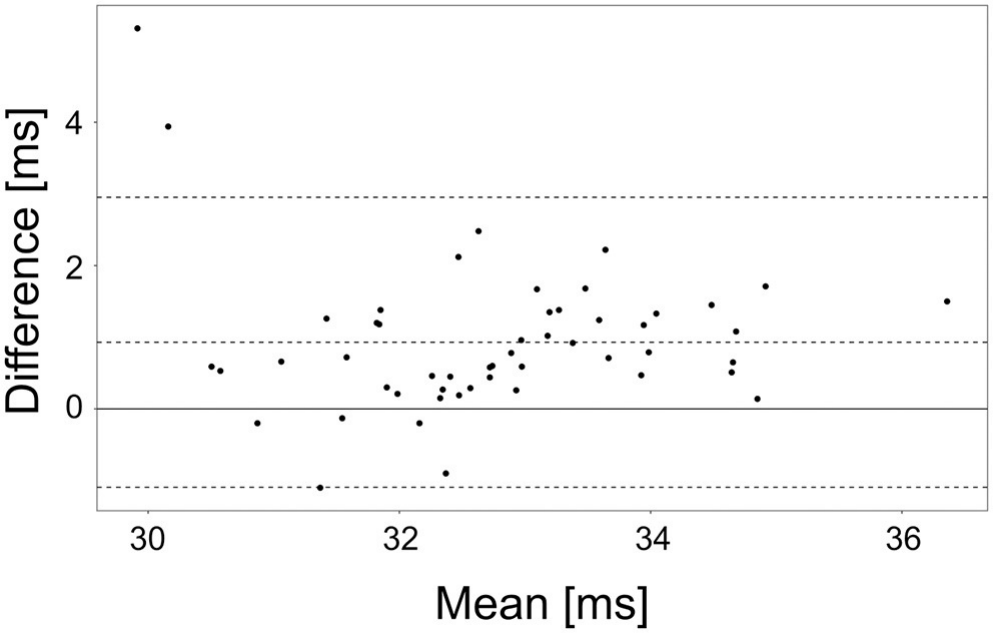
Bland Altman plot of the agreement of the wT2 values obtained by the QMRTools software package and the presented software, for an unconstrained 17-echo EPG reconstruction. The bias and the 95% confidence intervals are depicted in the plot.

The accuracy of the wT2 values was close to the QMRTools implementation, showing an average difference of 0.9 ms (5.2 ms standard deviation, Figure 5).

Concerning the fat fraction accuracy, although it is not the primary focus of this method, the EPG matching resulted in consistent underestimation compared to the FattyRiot algorithm, with a larger number of echoes providing results which were closer in average to the gold standard (from −4.2 percentage points (p.p.) for 17 echoes to -7.8 p.p. for eight echoes). The double exponential fitting provided an overestimation ranging from +3.2 p.p. for 17 echoes to +8.6 p.p. for five echoes.

## Discussion

In this work, we presented a software application that can quickly and reliably calculate wT2 maps in the presence of spectrally inhomogeneous voxels containing both non-fatty tissue and lipids, while at the same time estimating fat fraction from conventional multi-echo spin-echo acquisitions. This work follows the concept introduced by Marty et al. (14). Still, it additionally provides the possibilities of performing double exponential (and, optionally, single exponential) fitting and, more interestingly, of incorporating external fat fraction information to improve the accuracy of the fitting. There are no specific hardware requirements for this program; however, for better performance, a reasonable Cuda-compatible graphic card should be used. Consumer-grade GPUs deliver good performance, but more extensive dictionaries than the one tested in this current setup require increased memory on the device. The code is released with a free open source license and, in contrast to existing available implementations (36), this software package is exclusively based on free software and is platform-independent. This implementation can thus be considered ready to be widely used in a clinical research context and as a reference by other researchers.

In the comparison with an existing implementation, the absolute wT2 values obtained by this method are close, but not identical, to the ones obtained from QMRTools, although both methods are based on the same conceptual framework. One explanation could be the usage of different metrics while performing the dictionary matching. This finding highlights the necessity of having consistent acquisition and data processing pipelines, and the necessity of characterizing a quantitative method primarily in terms of reproducibility and precision. It is generally true also in other fields of quantitative MRI, that the absolute values obtained are method-dependent and thus comparisons need to be carefully considered (37, 38).

During the optimization of the EPG matching algorithm, it appeared clear that an accurate slice profile was very important to obtain absolute values of wT2 close to the literature. Small changes to the profile, or selecting a too large refocusing slice width factor, could introduce a bias of a few milliseconds in the estimated values. The intersubject and intrasubject variabilities, however, minimally changed, so the user should be advised to obtain the exact sequence characteristics and to use a coherent parameter set when reconstructing multiple datasets.

The introduction of the constrained reconstruction appears beneficial for the fitting of wT2, providing reliable and homogeneous results, not correlated with the amount of infiltration of the muscle, with considering as few as five acquired echoes. This is a valuable result in light of providing better spatial coverage by the MESE sequence: reducing the number of acquired echoes allows exciting multiple interleaved slices in a simple repetition time and thus lessen the interslice gaps. However, the alignment of the fat fraction map can introduce some artifacts if patient motion occurs between the two acquisitions. In the current implementation, no image registration is performed. The alignment of the MESE and MEGE datasets is currently only performed based on the orientation information provided in the image headers. Image registration is, however, implementable using free python libraries and could be added if needed.

The EPG matching, as expected, could account for the B1 inhomogeneities and therefore lead to lower artifacts in the areas where flip angles deviate from the nominal value in comparison to double-exponential fitting.

In general, unconstrained double exponential fitting with reduced echoes resulted in underestimating wT2 values relative to other methods with unconstrained reconstruction. On the other hand, thanks to its fewer degrees of freedom in the fitting, it showed remarkable homogeneity across the various ROIs even with few echoes, suggesting that it might still be considered a robust and straightforward approach when no other methods are available. The constrained double exponential reconstruction, conversely, produced results farthest from the expected values (12, 14). An analysis of the data shows that the unconstrained double exponential fitting consistently overestimates the fat fraction. The possible explanation is that the longer exponential decay due to the stimulated echoes gets assigned to the fat component during the fitting process in the unconstrained case, whereas fixing the fat fraction in the constrained reconstruction produces a longer apparent exponential decay constant in what is practically a monoexponential fitting of the residuals.

Generally, most of the obtained wT2 values negatively correlate with fat fraction, which is in line with the previous findings (15, 39), with the exception of the EPG matching of the reduced echoes, showing a positive correlation, suggesting a failure to separate the wT2 from fat.

As an overall comparison, both fitting methods (double exponential fitting and EPG matching) have advantages and disadvantages. EPG matching appears accurate and precise even when a lower number of echoes is used, especially when paired with an external fat fraction constraint, and it has a higher insensitivity to B1 inhomogeneity, but it requires precise knowledge of the acquisition parameters to obtain unbiased values. Double exponential fitting fails in regions of B1 inhomogeneity, its accuracy is poor when few echoes are used, and the external fat fraction constraints introduces a bias in the obtained values. However, it requires very little knowledge of the acquisition parameters, and it can therefore be chosen when the sequence characteristics are unknown. When the full 17-echo acquisitions are used, the two fitting methods have similar characteristics when averaged across the muscles; however, the sensitivity of the double exponential fitting to B1 inhomogeneity might mask local intramuscular changes in patients with neuromuscular disorders.

Although not explored in the present validation for the results to be more comparable, the program assumes a constant value for fat T2. Still, it gives the possibility of indicating it as a runtime parameter or estimating it from the image itself. Similarly, T1 values for water and fat are fixed, although this can be assumed to have a small effect on the final result (14). Other effects like j-coupling are also not explicitly introduced in the model but rather incorporated in the assumed value of fat T2, which is dependent on the characteristics of the MESE sequence (40).

The implementation has some further limitations regarding the accuracy of the results. Specifically, Keene et al. demonstrated that this method can be improved by introducing a correction for the chemical shift in the slice profile and a better estimation for fat T2 (15). These corrections are relatively newly introduced and not routinely used in the current studies, and thus not currently implemented. However, the chemical shift correction requires knowledge of the actual implementation of the pulse sequence that might be difficult to obtain in a clinical setting. It would therefore result in potential loss of generalizability. Similarly, a multi-peak spectral model for the fat could be incorporated into the algorithm, which could improve the results' accuracy. As this method is based on multi spin echoes, the multiple spectral peaks do not result in different chemical shifts as in gradient-echo images. The effect would only be seen in the different T2 values of the fat components; therefore, this functionality is not currently implemented, in line with existing spin-echo-based methods for muscle imaging (14, 15, 17, 23).

One limitation of this work is the relatively small number of datasets. However, both healthy and different diseased subjects were included. While a larger subject cohort would be of interest, one of the goals of this study was to offer the tools for such studies and not to draw conclusions on wT2 measurements of dystrophic muscles. For this goal, clinical studies that focus on more homogenous patient cohorts (grouping, for example, the different stages of neuromuscular disease, where similar pathological changes are expected) are required.

The validation of this work was performed by comparing the proposed fitting with an existing implementation of the same concept on the same data, thus lacking an external reference standard for the values obtained. While such an external reference could be desirable, the scope of this work was not to assess the validity of MESE acquisitions for the estimation of wT2, but only to evaluate the efficacy of the proposed implementation. The availability of an independent fitting procedure allowed a direct comparison of these characteristics without other sources of error deriving from the physics of different acquisition methods.

In conclusion, in this work, we have presented a fast and open implementation of an algorithm for the T2 mapping of the water component of muscle tissues in the presence of fat, based on conventional multi-echo spin-echo sequences, capable of incorporating prior knowledge of the fat fraction. Thanks to the additional information obtained from the multi-echo gradient-echo images, a reduced number of echoes can be used for the spin-echo acquisition with while retaining similar inter- and intrasubject variability and similar absolute values, when and EPG model is used. The EPG matching method is in general to be preferred to a double exponential fitting, provided that the characteristics of the MR sequence (especially in terms of RF pulses) are sufficiently known.

## Data Availability Statement

The data analyzed in this study is subject to the following licenses/restrictions: Data included in this work can be shared upon research agreement between the Institutions. Requests to access these datasets should be directed to Anna Pichiecchio, anna.pichiecchio@mondino.it.

## Ethics Statement

The studies involving human participants were reviewed and approved by Comitato Etico Area Referente Pavia Fondazione IRCCS Policlinico San Matteo and Policlinico Gemelli's Ethics Committe. The patients/participants provided their written informed consent to participate in this study.

## Author Contributions

FSa study conception, method and software development, data analysis, and manuscript drafting. XD data analysis, statistics, and manuscript drafting. MP, FSo, and AP data acquisition and patient management. MW and PS method development. MM, ER, and GT patient recruitment and clinical evaluations. OB, AP, and NB study conception and supervision. All authors contributed to the article and approved the submitted version.

## Conflict of Interest

The authors declare that the research was conducted in the absence of any commercial or financial relationships that could be construed as a potential conflict of interest.
